# Cardiopulmonary rehabilitation in post-COVID-19 patients: case series

**DOI:** 10.5935/0103-507X.20210018

**Published:** 2021

**Authors:** Cláudia Tozato, Bruno Fernandes Costa Ferreira, Jonathan Pereira Dalavina, Camila Vitelli Molinari, Vera Lúcia dos Santos Alves

**Affiliations:** 1 Physiotherapy Service, Irmandade da Santa Casa de Misericórdia de São Paulo - São Paulo (SP), Brazil.; 2 Instituto de Assistência Médica ao Servidor Público Estadual “Francisco Morato de Oliveira” - São Paulo (SP), Brazil.; 3 Medical Sciences College, Irmandade da Santa Casa de São Paulo - São Paulo (SP), Brazil.; 4 Professional Program in Health Science and Technology – Master’s Degree, Universidade de Mogi das Cruzes - Mogi das Cruzes (SP), Brazil.

**Keywords:** Coronavirus infections, COVID-19, Physical therapy, Rehabilitation, Walk test, Dyspnea, Fatigue, Muscle strength, Infecções por coronavírus, COVID-19, Fisioterapia, Reabilitação, Teste de caminhada, Dispneia, Fadiga, Força muscular

## Abstract

The natural history of the disease, and the treatment of post-COVID-19 patients, are still being built. Symptoms are persistent, even in mild cases, and the infection consequences include fatigue, dyspnea, tachycardia, muscle loss, and reduced functional capacity. Regarding cardiopulmonary rehabilitation, there seems to be an improvement in functional capacity, quality of life, and prognosis with the 6-Minute Walk Test used as a prognostic and therapeutic evaluator. Therefore, this case series report aims to present our experience with four cases of different severity levels, involved in a post-COVID-19 cardiopulmonary rehabilitation program. These patients were assessed with the 6-Minute Walk Test, peripheral muscle strength, and double product at rest, to assess the results after a three-month rehabilitation protocol of at least 300 minutes per week. The four patients had their distance covered during the walk test increased between 16% and 94%. Peripheral muscle strength was improved by 20% to six times the baseline values, and double product at rest was reduced by 8% to 42%. The cardiopulmonary rehabilitation program had a positive impact on these cases, improving functional capacity despite the different severity levels in these post-COVID-19 cases.

## Abstract (pt)

A história natural da doença e o tratamento de pacientes após a COVID-19 ainda se apresentam em construção. Os sintomas são persistentes, mesmo em casos leves, e as consequências decorrentes da infecção incluem fadiga, dispneia, taquicardia, perda de massa muscular e diminuição da capacidade funcional. Sobre a reabilitação cardiopulmonar, parece haver melhora na capacidade funcional, na qualidade de vida e no prognóstico com o Teste da Caminhada de 6 Minutos, sendo este utilizado como avaliador prognóstico e terapêutico. Assim, o objetivo deste relato de casos é descrever a experiência de quatro casos, de diferentes gravidades, que realizaram um programa de reabilitação cardiopulmonar pós-COVID-19, avaliados com Teste da Caminhada de 6 Minutos, força muscular periférica e duplo produto em repouso, para verificar o efeito da reabilitação após 3 meses de protocolo de, no mínimo, 300 minutos por semana. Os quatro casos apresentaram aumento da distância percorrida no teste da caminhada entre 16% e 94%. Houve aumento da força muscular periférica em 20% até seis vezes seu valor inicial, e a redução do duplo produto em repouso variou entre 8% e 42%. O programa de reabilitação cardiopulmonar apresentou impacto positivo nos casos acompanhados, com melhora da capacidade funcional, mesmo com a variabilidade da gravidade dos casos pós-COVID-19.

## INTRODUCTION

The coronavirus disease 2019 (COVID-19) is known to cause acute respiratory failure, with cardiopulmonary changes that were not yet fully clarified, featuring severe manifestations in up to 67% of the hospitalized patients with acute respiratory distress syndrome, characterized by severe hypoxemia requiring oxygen therapy and supportive measures.^(1,2)^ Treatment and progression of these cases are still to be fully understood, given the, so far, little knowledge on this disease’s natural history.

Post-COVID-19 symptoms are persistent, even for mild cases.^(2-4)^ Its consequences include fatigue, dyspnea, tachycardia, muscle loss, and reduced functional capacity. Studies^(3-6)^ have shown that cardiopulmonary rehabilitation (CPR) can improve patients’ functional capacity, quality of life, and prognosis.

Currently, there is little information available in the literature to customize the rehabilitation following COVID-19 infection or hospitalization.

This case series is aimed to present our experience with different severity levels post-COVID-19 patients, who were included in an CPR program for three months.

## CASE REPORTS

This series reports on the rehabilitation of four post-COVID-19 patients with varying degrees of severity and trained according to the protocol shown in table 1. All patients signed an Informed Consent Form, and the project was approved ty the *Irmandade da Santa Casa de Misericórdia de São Paulo*’s Research Ethics Committee under the registration number CAAE 33118220.8.0000.5479.

**Table 1:** Cardiopulmonary rehabilitation protocol for post-COVID-19 patients

Protocol	Cardiopulmonary rehabilitation - COVID-19
Aerobic exercise	Treadmill, upper and lower limbs cycle ergometer, and step exercises
Load	60% and 80% of HR reserve (Karvonen) Borg Scale (0 - 10) between 4 and 6, SpO2 ≥ 90%
Volume	3 times/week, 30 minutes
Resistance exercise	1MR test
Load	Weekly assessment 60% MR, all muscle groups
Volume	3 times/week 3 series 10 repetitions each
Baseline and after 3 months	6-Minute Walk Test, handgrip strength test, and 1MR test for each muscle group

HR - heart rate; SpO_2_ - peripheral oxygen saturation; MR - maximal repetition.

### Case 1

Female, 57 years old, with a history of hypertension treated with amlodipine. Presented on June 3, 2020, with flu-like symptoms and reduced peripheral oxygen saturation (SpO_2_). Arterial blood gas examination showed mild hypoxemia and her COVID-19 swab tested positive. She was discharged and instructed to maintain social isolation and leave work. One hundred and seven days after the symptoms start, she was referred to cardiorespiratory physical therapy for dyspnea to medium exertion. Since then, she has been the rehabilitation program.

### Case 2

Male, 72 years old, with a history of hypertension, smoking, HIV, and a prostate cancer treated with radiotherapy. Taking losartan, amlodipine, and antiretroviral therapy. He was admitted to the emergency department with flu-like symptoms, reduced oxygenation, chills, and diarrhea for five days. Denied having dyspnea, fever, or cough. He was admitted to the hospital on June 4, 2020, undergoing oxygen therapy by a nasal catheter 2L/min (SpO_2_ 98%), and had a COVID-19 positive swab. Chest computed tomography (CT) scan showed 50% involvement of the lung area. The patient was given antibiotic therapy, amlodipine, and enoxaparin. During the first five days, he underwent oxygenation with a non-rebreather oxygen mask with a reservoir bag, up to 5L/minute, followed by weaning. He was hospitalized for seven days and was referred to rehabilitation for dyspnea.

### Case 3

Male, 52 years old, history of hypertension, admitted to the emergency department on July 14, 2020, with tachypnea, tachycardia, and SpO_2_ 72% on room air. He had dyspnea at rest, dry cough, and fever for four days. Admitted to the hospital, received oxygen therapy with a non-rebreather mask with a reservoir bag, at 10L/minute. A chest CT scan showed more than 50% involvement of the lungs; the swab test was positive for COVID-19. On the same day, he was referred to the intensive care unit due to worsened respiratory condition. He was treated for nine days with drugs, oxygen therapy, non-invasive ventilation, spontaneous prone positioning, and early mobilization by the physical therapy team. He was discharged from the hospital after a 9-day hospitalization and referred to cardiorespiratory physical therapy for dyspnea and fatigue.

### Case 4

Female, 43 years old, previously healthy. On March 15, 2020, she had headache, dry cough, nasal obstruction, and fever (38.5ºC), sought medical attention and was given antibiotics. On March 21, 2020, she had worsened symptoms with exertion dyspnea, and a chest CT scan showing ground-glass signs and was recommended social isolation. On March 29, 2020, she went back to seek medical attention, with severe diarrhea. A new chest CT scan showed bilaterally worsened signs with a 50% involvement, requiring hospitalization. On April 5, 2020, she was intubated, had difficult weaning, and underwent tracheostomy. During the hospitalization, she underwent hemodialysis for 16 days. Discharged from the hospital on May 20 after an about two-month hospitalization, she had tetraparesis, was restricted to a wheelchair, had an occluded tracheostomy, and her COVID-19 test was negative. She was referred to rehabilitation for neuromuscular and cardiorespiratory deficits. Due to tetraparesis, she was assessed for inspiratory muscle strength and started training with POWERbreathe with weekly adjustments, at 30% maximal inspiratory pressure. As the mobility progressed, started training with cycle ergometer for upper and lower limbs, in association with resistance training, until independent walking was achieved.

The protocol results after a three-month CPR follow-up are shown in table 2; there was cardiovascular recovery as assessed by the double product, reduced exertion dyspnea, increased peripheral muscle strength, and functional independence as reported and observed throughout the rehabilitation. The 6-Minute Walk Test (6MWT) covered distance was increased by 16%, 49%, 67%, and 94% for cases 1 to 4, respectively; double product, was reduced by 42%, 27%, 8%, and 34% for cases 1 to 4, respectively. Borg scale-associated dyspnea variables were reduced for all cases, showing increased functional capacity and improved prognosis, irrespective the disease severity.

**Table 2:** Assessment and reassessment of functional capacity and peripheral muscle strength in the four reported cases

TC6M	Case 1		Case 2		Case 3		Case 4		
	**Baseline**	**3 months**	**Baseline**	**3 months**	**Baseline**	**3 months**	Baseline wheelchair*	**45 days First 6MWT**	3 months* after the first 6MWT
Maximal HR (bpm)	154	140	120	155	123	132	125	128	164
Minimal SpO_2_ (%)	95	94	91	96	89	94	97	97	97
Covered distance (m)	490	570	364	543	430	718	MaxIP: 80cmH_2_O	300	583
Maximal Borg	6	3	7	4	2	0	6	4	5
Double product (HR x SBP) rest	12,240	8,580	11,640	9,130	13,910	12,870	12,320	10,800	9,200
MR knee extension	14 R	21 R	7 R	21 R	7 R	14 R	0 R	1 R	6 R
	14 L	21 L	7 L	21 L	7 L	14 L	0 L	1 L	6 L
MR shoulder abduction (kg)	1.5 R	2.5 R	2 R	3 R	2 R	3 R	0 R	0.5 R	2 R
	1.5 L	2 L	2 L	2.5 L	1.5 L	3 L	0 L	0.5 L	2 L
MR elbow flexion (kg)	2.5 R	3 R	1 R	2.5 R	3 R	5 R	0 R	1.5 R	4 R
	2.5 L	3 L	1 L	2.5 L	2.5 L	4 L	0 L	1.5 L	4 L
Handgrip (kg)	19 R	26 R	25 R	26 R	29 R	31 R	0 R	8.7 R	24.7 R
	20 L	25 L	19 L	21 L	26 L	31 L	0 L	9.4 L	23.7 L

6MWT - Six-minute Walk Test; HR - heart rate; SpO_2_ - peripheral oxygen saturation; MIP - maximal inspiratory pressure; SBP - systolic blood pressure; MR - maximal repetition; R - right side; L - left side.

* 4 1/2 months in total.

In all cases, the peripheral muscle strength was increased, ranging from 20% to six times the baseline values. However, for the palmar grip, in cases two and three a wrist tendinopathy and late left humerus fracture were observed. The fourth case required differentiated care, with cardiopulmonary physical therapy, neurofunctional and occupational therapy, which, in association with medical attention, allowed all walking aids to be removed. The results show that a customized rehabilitation allowed the achievement good results in this case series.

## DISCUSSION

This study presented four cases with different COVID-19 severity degrees and outcomes. The rehabilitation program was based on cardiovascular and pulmonary rehabilitation principles, with an emphasis on possible pulmonary sequelae including reduced SpO_2_ and dyspnea. Desaturation was found in two cases, and all patients reported dyspnea during the workout. Functional capacity and peripheral muscle strength reduction were approached by rehabilitation programs, and the 6MWT is recommended as a tool to assess exertion limitations and training prescription, as well as for reassessment and prognosis.^(7-9)^

Recently, a study was published regarding the use of 6MWT for the assessment of post-COVID-19 silent hypoxemia, with a thromboembolic event diagnosis by the time of the discharge.^(10)^ In this series, only the second and third cases had reduced SpO_2_ during the first 6MWT; however, the fourth case presented a right lower limb edema during the rehabilitation process and was diagnosed a non 6MWT-associated right iliac vein thrombosis, and given drug therapy.

The 6MWT is the most frequently used submaximal exertion test in pulmonary^(8)^ and cardiac^(9)^ rehabilitation, however, patients hospitalized for COVID-19 may have test preventing mobility impairments, as our fourth case, where the woman was tetraparetic and restricted to a wheelchair. Alternatively, we used inspiratory muscle training in association with other physical therapy specialties. Upon progression of mobility, we started resistance and aerobic training with a cycle ergometer for the upper and lower limbs. This approach was initially used in China, according to the first publications involving post-COVID-19 patients, as a daily-use home tool, with good results.^(4)^

Progressively the cases benefited from aerobic and resistance training as tested with the one maximal repetition (1MR). After three months, symptoms were reduced and the distance covered during the 6MWT was increased, as well as the peripheral muscle strength; similar results are reported in post-COVID-19 patients’ Chinese papers.^(4,5)^ The improvement found in these short-term protocols may be related to the disease’s natural clinical progression. However, previous studies in patients after acute respiratory distress syndrome have shown permanent sequelae, never reaching the predicted distance in the 6MWT, even five years after the initial condition.^(7)^

The lack of pulmonary function and inspiratory muscle strength assessment, cardiopulmonary and ergometric testing, quality of life questionnaires, or assessment of the functional capacity by other means in all cases, are limitations of this report. However, these limitations did not prevent assistance to these patients, taking into consideration the pillars of the CPR and achieving good results. The treatment site required care with the risk of contamination, equipment spacing, reduction of the number of patients per session, constant local hygiene, and identification of symptoms with possible removal, to allow starting the rehabilitation process in this profile of post-COVID-19 patients as soon as possible. Studies on the rehabilitation of post-COVID-19 patients are still few. As this is a relatively recent disease, its treatment process is still being built.^(2-6)^

## CONCLUSION

The physical exercise program based on principles of cardiovascular and pulmonary rehabilitation had a positive impact on this case series, showing improved functional capacity despite the variability in the severity of these post-COVID-19 patients.
